# Uterine fibroid embolization: An analysis of clinical outcomes and impact on patients’ quality of life

**DOI:** 10.1515/med-2025-1235

**Published:** 2025-08-07

**Authors:** Nicolò Ubaldi, Marcello Andrea Tipaldi, Andrea Giannini, Tullio Golia D’Augè, Donatella Caserta, Antonio Simone Laganà, Giacomo Grasso, Aleksejs Zolovkins, Edoardo Ronconi, Michele Rossi

**Affiliations:** Department of Surgical and Medical Sciences and Translational Medicine, Azienda Ospedaliera Sant’Andrea, Sapienza University of Rome, Rome, Italy; Department of Interventional Radiology, Sant’Andrea Hospital, University La Sapienza, Rome, 00189, Italy; Department of Gynecological, Obstetrical and Urological Sciences, “Sapienza” University of Rome, Rome, Italy; Department of Maternal and Child Health and Urological Sciences, Policlinico Umberto I, Sapienza University of Rome, Rome, 00161, Italy; Unit of Obstetrics and Gynecology, Paolo Giaccone Hospital, Department of Health Promotion, Mother and Child Care, Internal Medicine and Medical Specialties (PROMISE), University of Palermo, 90127, Palermo, Italy

**Keywords:** uterine artery embolization, uterine fibroid, quality of life, symptom severity

## Abstract

**Background:**

Uterine fibroids (UF) affect up to 70–80% of women by age 50 and are associated with heavy menstrual bleedings, pelvic discomfort, and reduced quality of life. Uterine artery embolization (UAE) is a minimally invasive procedure that aims to reduce fibroid-related symptoms and improve patients’ quality of life.

**Materials and methods:**

A prospective single-center study was conducted on 40 women who underwent UAE between November 2018 and June 2023. Primary outcomes were evaluated using the Uterine Fibroid Symptom and Quality-of-Life (UFS-QoL) questionnaire to assess symptom severity and health-related quality-of-life scores. Secondary outcomes include pain intensity, complications, requirement of additional treatments, and duration of symptoms after discharge.

**Results:**

Thirty-three patients completed the follow-up UFS-QoL questionnaire. The mean age and the mean follow-up time were 47 years (SD 14 years) and 10 months (SD 4 months), respectively. Substantial improvements were observed within all domains of the UFS-QoL questionnaire (*p* < 0.001), particularly in symptom severity, where 94% of patients experienced a benefit. All Health Related Quality-of-Life (HRQL) domains statistically significantly improved (73–91%; *p* < 0.001). 67% of patients did not require further treatments. Pelvic pain, according to VAS, improved by 2.1 (0 = much better; 5 = no change; 10 = worse). Post-procedural complications were minor, 70% of patients reported symptoms persisted up to 5 days after discharge, and 88% resolved within 2 weeks. Despite a 45.5% rate of minor complications, including pain and transitory bleeding, 88% of patients would recommend UAE, underlining its safety and effectiveness.

**Discussion and conclusion:**

UAE offers substantial benefits for symptomatic fibroids, significantly improving HRQoL, symptom severity and pelvic pain scores, making it a valuable alternative to surgery. Complications were minor and short-lived, and the majority of patients were satisfied with the results, with no need for additional treatments. Further research is warranted to generate peri-procedural pain management consensus guidelines, clinical outcomes of radial access, and fertility-related outcomes.

## 1 Introduction

Uterine fibroids (UF), the most common benign tumors in reproductive-aged women, affect 70–80% of women by age 50 and significantly impact the quality of life through symptoms such as menorrhagia, pelvic pain, and fertility challenges [1,2,3,4]. Many fibroids regress post-menopause; thus, observant waiting is viable for perimenopause patients with manageable symptoms. Despite the availability of various treatments, the global need for minimally invasive alternatives to surgery remains unmet.

Uterine artery embolization (UAE), first described in 1995, has emerged as a minimally invasive, valuable option, but data on its long-term efficacy and global applicability are limited [5]. Arterial access is typically through the common femoral artery or left radial artery, the latter being associated with improved post-procedural pain management [6]. Some prefer bilateral femoral access for embolizing both uterine arteries, which is theoretically correlated with a reduced time of the procedure [7]. Notably, UAE shares technical principles with hemorrhoidal artery embolization, a minimally invasive technique that has gained attention for its promising outcomes in treating hemorrhoidal disease [8]. Both procedures rely on selective arterial embolization to reduce vascular supply to the pathological tissue, leading to symptom relief and long-term control. The technical success rate of UAE is very high (>95%), depending on the tortuous anatomy, leading to a measurable elimination of abnormal uterine bleeding associated with fibroids in over 90% of treated women, a substantial improvement in subjective mass symptoms, and an 80–90% satisfaction rate among treated women [9,10].

Compared to surgical alternatives, UAE involves less blood loss, shorter hospital stays, less expensive procedures, and shorter recovery time [11]. The “FEMME” trial, which randomized 127 patients to myomectomy and 127 to UAE, assessed health-related quality of life using the Uterine Fibroid Symptom and Qualityof-Life (UFS-QoL) questionnaire [12]. At the two-year mark, myomectomy showed significantly better quality of life (*p* = 0.01), also related to the higher re-intervention rate associated with UAE (15–32% compared to 7% for surgery), despite a higher complication rate and longer hospital stays. However, by 4 years, there were no significant differences in quality of life between the two groups, and pregnancy rates were comparable for both treatments. A lower rate of complications may balance out the initial cost-benefit advantages of UAE over surgical treatment [11].

This study aims to assess the effectiveness of UAE treatment on health-related quality of life (HRQL) and symptom severity, according to the UFS-QoL questionnaire, along with other secondary outcomes, in patients with symptomatic UF.

## 2 Materials and methods

### 2.1 Study design

A prospective single-center single-cohort study was conducted. Data were collected over 5 years, from November 2018 to June 2023, involving a cohort of 40 patients with uterine fibromatosis, who underwent UAE at the Interventional Radiology Unit of Sant’Andrea Hospital in Rome, Italy. The diagnosis and evaluation of UF in women aged 25–55 years with symptomatic fibroids typically involves a thorough medical history, pelvic examination, and magnetic resonance imaging. Exclusion criteria encompassed pelvic infections or inflammatory disease, active cancer, pregnancy, significant adenomyosis, or contraindications to UAE. The mean age was 50 years ±11, the median parity was 1, the location of the largest fibroid was 2 (5%) in the submucosa, 8 (20%) in the subserosa, 27 (67.5%) in the muscle wall, and 3 (7.5%) were missing. The largest fibroid was <7 cm in 17 (42.4%) and >7 cm in 23 (57.5%), and the mean was 9 cm ± 3 cm. Follow-up was conducted in the time interval between 6- and 12-month post-procedure, assessing symptom severity and HRQL using the UFS-QoL questionnaire and the visual analogue scale (VAS). All methods or experimental protocols were approved by the local Institutional Review Board, and data were collected in accordance with the 1964 Helsinki declaration and its later modifications.

### 2.2 Outcome measures

The primary outcome was to compare the quality of life and symptom severity before UAE and at least 6 months after UAE, using the UFS-QoL questionnaire. The UFS-QoL questionnaire, developed by Spies et al. [13], is a validated 37-question survey designed to evaluate the severity of symptoms and health-related quality of life in patients with UF. The first eight questions are related to the domain of “Symptom Severity,” whilst the remaining 29 questions are related to the other six domains grouped under HRQL. Quality of life was assessed using the validated UFS-QoL questionnaire, which includes symptom severity and HRQoL domains. Raw scores were summed within each domain and transformed into a 0–100 scale, following the standardized scoring procedure. Higher scores indicate greater symptom severity (Symptom Severity Scale) and better quality of life (HRQoL subscales). No weighting was applied to individual domains. Score interpretation followed standard guidelines established in previous validation studies.

Other secondary outcomes were evaluated using a follow-up questionnaire, which included additional questions on pregnancies and spontaneous abortions before and after treatment. The questionnaire also assessed procedural characteristics such as complications, improvement in pelvic pain intensity (0–10 VAS scale), duration of symptoms after discharge, the need for additional treatments, and overall satisfaction with the treatment. The Numerical Pain Rating Scale (NPRS) was assessed in the short-term period in the hospital post-procedure.

### 2.3 Statistical analysis

Data were analyzed using SPSS version 27. Normality was tested with Kolmogorov–Smirnov and Shapiro–Wilk tests, followed by Wilcoxon signed-rank tests for non-parametric comparisons and chi-squared tests for categorical variables.

## 3 Results

### 3.1 Population

**Figure 1:**
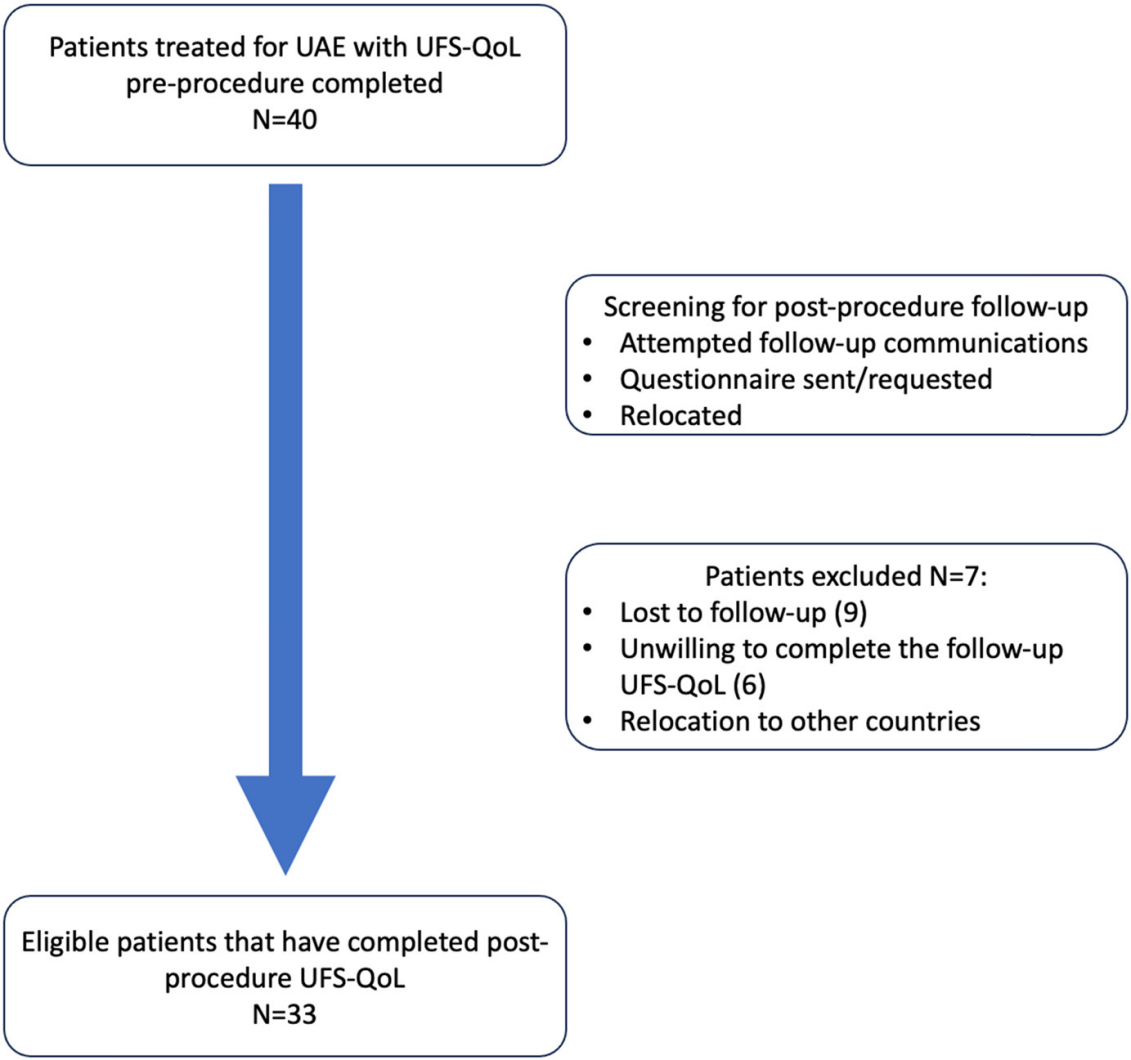
Flowchart of the study population selection.

Out of the initial cohort of 40 patients, 7 patients were lost, leaving 33 patients who completed the UFS-QoL questionnaire before and after the procedure (Figure 1). Reasons for loss to follow-up included lack of response to follow-up communications, refusal post-procedure questionnaire completion and relocation. No specific patterns regarding baseline characteristics (such as age, baseline fibroid size, or symptom severity) were identified among those lost to follow-up compared to the overall study population. The mean follow-up time was 10 months (SD 4 months). In the final population, the mean age was 47 years ±14 (SD 6), and the majority of the women (85%) were in pre-menopause. The mean size of the largest fibroid was 8 cm ± 3.0, and the median IQR of the number of fibroids was 3 (1–5) (Table 1).

**Table 1:** Baseline characteristics of the analyzed population UAE (*N* = 33)

Characteristic	UAE (*N* = 33)
Age, year	47 ± 14 (SD 6)
Parity median (mean)	0.0
Gravidity median (mean)	0.0
**Fertility Status**	
Pre-menopause	28
Post-menopause	5
**Location of largest fibroid with MRI – no. (%)**	
Submucosa	1 (3.0%)
Subserosa	10 (30.3%)
Muscle wall	22 (66.7%)
Data missing	0 (0%)
**Largest dimension of largest fibroid – no. (%)**	
≤7 cm	13 (39.4%)
>7 cm	20 (60.6%)
Mean, cm	8 ± 3.0
**No. of fibroids, no. (%)**	
1–3	13 (39.4%)
4–10	9 (27.3%)
>10	11 (33.3%)
Median (IQR)	4 (1–5)

### 3.2 Primary outcome results

The raw domain-specific scores assessed by the UFS-QoL questionnaire are presented in Table 2. UFS-QoL scores are provided in percentages for each domain and the HRQL TOTAL score. Higher scores in “Symptom Severity” indicate greater symptom intensity, while higher scores in HRQL domains indicate better health-related quality of life.

**Table 2:** Raw pre- and post-treatment scores by the UFS-QoL questionnaire

Domain	Score
Symptom severity before	66
Symptom severity after	26
Concern before	81
Concern after	33
Activities before	40
Activities after	82
Energy before	38
Energy after	79
Control before	43
Control after	87
Self-conscious before	61
Self-conscious after	86
Sexual function before	40
Sexual function after	75
HRQL-total before	40
HRQL-total after	81

Using the Wilcoxon signed-rank test, pre- and post-treatment questionnaire scores for each domain were compared, and all domains showed significant improvements (Table 3):

– Negative ranks indicate higher pre-treatment scores, compared to post-treatment scores, suggesting symptom reduction.
–Positive ranks indicate higher post-treatment scores, compared to pre-treatment scores, suggesting improved quality of life.
– Even indicate unchanged scores.

**Table 3:** UFS-QoL outcomes presented according to positive rank and negative rank scores

		*N*	Average rank	Sum of the ranks
Symptom severity after – symptom severity before	Negative rank	31	17.70	550
	Positive rank	2	5.50	11
	Even	0		
	Total	33		
Concern after – concern before	Negative rank	2	1.75	3.50
	Positive rank	26	15.48	402.50
	Even	5		
	Total	33		
Activities after – activities before	Negative rank	0	0	0
	Positive rank	28	14.5	406
	Even	5		
	Total	33		
Energy after – energy before	Negative rank	0	0	0
	Positive rank	30	15.5	465
	Even	3		
	Total	33		
Control after – control before	Negative rank	2	1.75	3,5
	Positive rank	29	16.98	492.5
	Even	2		
	Total	33		
Self-conscious after – self-conscious before	Negative rank	2	3.75	7.50
	Positive rank	25	14.8	370.5
	Even	6		
	Total	33		
Sexual function after – sexual function before	Negative rank	3	7	21
	Positive rank	24	14.88	357
	Even	6		
	Total	33		
HRQL total after – HRQL total before	Negative rank	0	0	0
	Positive rank	33	17	561
	Even	0		
	Total	33		

The statistical analysis showed significant frequency variations in scores between pre- and post-treatment, with *p* < 0.001 for all comparisons (Table 4).

**Table 4:** Pre- and post-treatment UFS-QoL scores comparison

	Sy. after – Sy. before	Conc. after – Conc. before	A. after – A. before	E. after – E. before	Cont. after – Cont. before	Self. after – Self. before	Sex. after – Sex. before	HRQL after – HRQL before
*Z*	−4,815	−4,546	−4,623	−4,784	−4,794	−4,369	−4,050	−5,012
Sign. asint.	<0.001	<0.001	<0.001	<0.001	<0.001	<0.001	<0.001	<0.001

### 3.3 Secondary outcome results

Major complications were not observed. Adverse effects were managed conservatively with simple analgesia medications and eventually resolved in all cases.

Fifteen patients (45.5%) had adverse effects related to the UAE procedure: the most frequent were intense pain (11 patients), prolonged menstrual bleeding (2 patients), and fever (2 patients). Post-embolization syndrome (PES) was present in 39% of patients.

**Figure 2:**
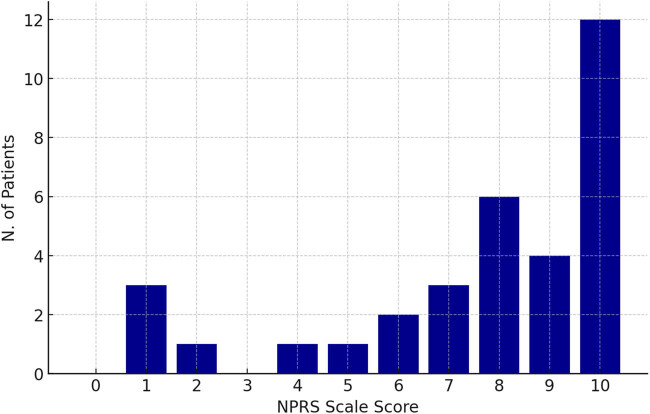
Pain score in the hours following the operation, measured on an NPRS scale.

The NPRS was performed during hospitalization to assess short-term post-procedural pain intensity. A majority of patients reported high pain scores during the initial hours following the procedure, with score 10 being the most common, affecting 12 patients (Figure 2). Moderate-high pain scores (7–9) were also frequently reported (13 patients). Lower-moderate pain scores (0–6) were less frequent (8 patients), with a small number of patients experiencing minimal or no pain.

**Figure 3:**
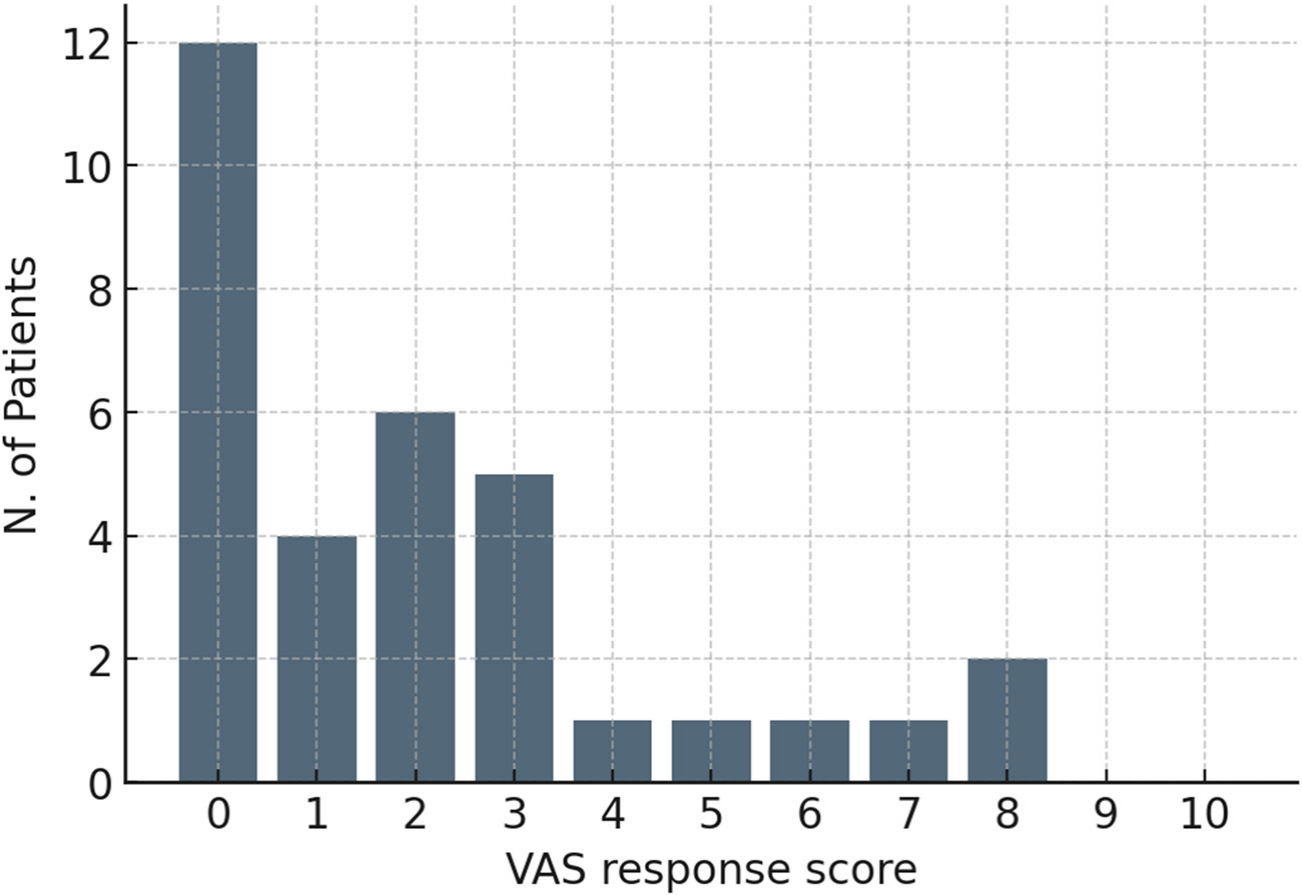
VAS Response scores after UAE.

According to VAS (0 = much better; 5 = no change; 10 = worse), patients reported an average pelvic pain improvement after treatment of 2.1 (Figure 3).

**Figure 4:**
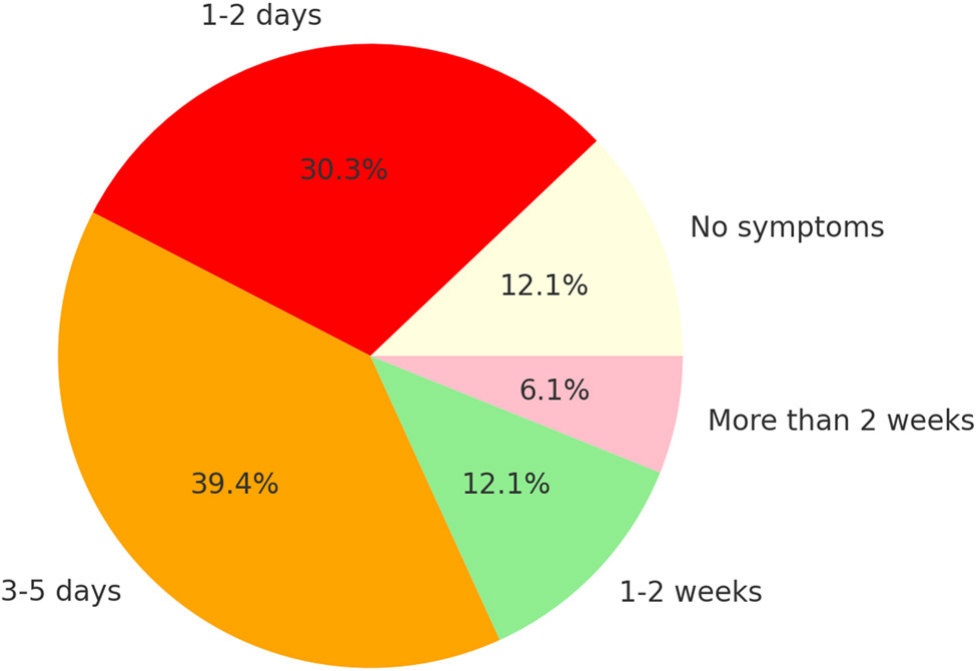
Duration of symptoms after UAE.

The duration of reported symptoms after hospital discharge varied among patients: 2 patients (6.1%) experienced no symptoms, 10 patients (30.3%) reported symptoms resolution within 1–2 days post-discharge, 13 patients (39.4%) reported symptoms resolution within 5 days, 4 patients (12.1%) reported symptom persistence for up to 2 weeks, and 4 patients (12.1%) experienced symptoms lasting longer than 2 weeks (Figure 4).

**Figure 5:**
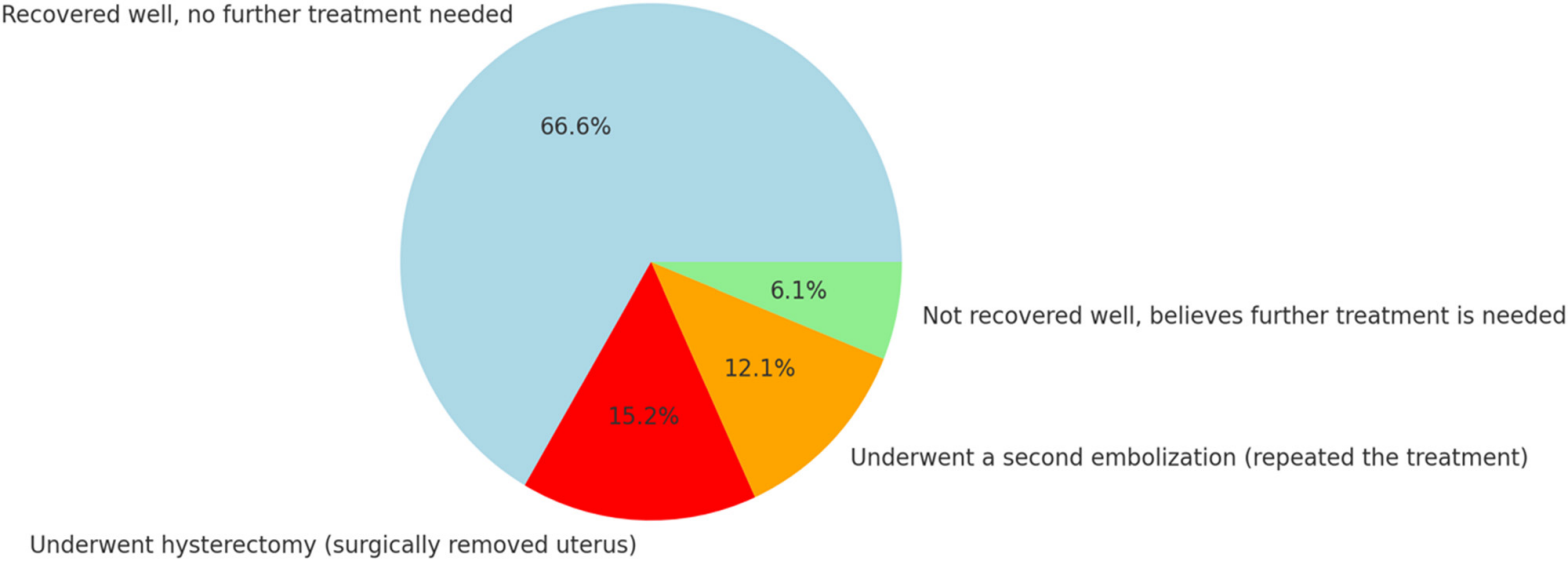
Number of patients who underwent re-treatment (UAE) or different therapies.

After the treatment, 22 patients (66.7%) reported feeling well and did not feel the need for further treatments; 2 patients (6.1%) reported having undergone hysterectomy; 5 patients (15.2%) reported having undergone a second UAE procedure; 4 patients (12.1%) believed they needed further treatments (Figure 5). 88% of patients (29 out of 33) would recommend this treatment to other women.

The most prevalent motivations for patients to undergo this type of treatment include word-of-mouth (24.2%), recommendation of their gynecologist (18.2%), and through Social Networks (15.2%).

## 4 Discussion

This study demonstrates the significant positive impact of UAE on symptom relief and health-related quality of life in patients with symptomatic UF, in alignment with existing literature [9,10,14,15]. Over the follow-up interval of 10 months, improvement in symptom severity and HRQL was observed in 94% and 73–91%, respectively; *p* < 0.001 for each comparison, according to the standardized UFS-QoL questionnaire. As previously reported, improvements in HRQL occur rapidly in the early stages and reach their maximum in the long term (>7 months) [16]. “Sexual function” showed the least improvement (73%), likely due to the limited assessment of psychological features, although studies focusing on longer follow-up have shown enhancements after 12 months [17,18]. In this study, the pelvic pain score improved substantially, ultimately resulting in a 2.1 VAS.

Pain is the most commonly reported symptom following UAE, with approximately 90% of patients experiencing postoperative pain, compared to 30% during the procedure [19]. Pain peaks within 6–8 h, gradually declines over 24 h, remains mild for 2–3 days, and typically resolves within 7–10 days [20]. Pain, often due to ischemic necrosis, was common, with 75.7% of patients reporting pain ≥7 on an NPRS scale, peaking at a score of 10 in 36.4% of cases. Despite these challenges, 70% of patients experienced symptom resolution within 5 days, and 88% recovered within 1–2 weeks, reinforcing the transitory nature of these complications [21]. When pain is accompanied by fever, nausea, headache, and fatigue, it is termed PES and can be present in 30–40% of the population [19,22]. In our study, transient complications were reported in 45.5% of cases, the most frequent being PES, present in 39% of cases.

UAE is a key treatment option for symptomatic fibroids in current clinical practice. These results confirm UAE’s validity as a minimally invasive alternative to surgical treatments, particularly for fibroids <15 cm, where considerable symptom relief can be achieved without residual compressive effects. There is a common perception that large fibroids (>10 cm) are associated with an increased risk of complications after UAE, such as infection and ischemic uterine injury; however, this was ultimately disproven [23,24]. However, other authors have reported better clinical response rates in patients with smaller fibroids [16].

Moreover, patients undergoing UAE typically recommence work and daily activities within two weeks, compared to six weeks for surgery [15]. Although 88% of patients expressed satisfaction with the procedure, 27% required additional treatment (12% repeat UAE and 15% hysterectomy), which is slightly higher than the rates reported in the literature (7–14% at 12 months and 24–27% at 5 years), likely due to our shorter follow-up interval, differences in procedural techniques and possibly different patient population baseline characteristics, such as larger (mean: 9 cm ± 3.0) and numerous fibroids (median IQR: 3 (1–5)) compared to the FEMME trial (mean: 7.6 cm ± 3.2; Median IQR: 2 (1–5), respectively) which documented a re-intervention rate of 16% [25,26,27,28]. This underscores the importance of thorough pre-procedural counseling regarding the risks of reintervention [29]. Despite these challenges, the lower complication rates and shorter hospital stays, compared to surgery, make the UAE a cost-effective option in most cases [11,12].

One of the main strengths of this study is its prospective design, which provides robust data on the clinical outcomes and patient satisfaction associated with UAE. The use of internationally validated tools, such as the UFS-QoL questionnaire, adds reliability to the findings. Furthermore, the focus on patient-reported outcomes highlights the procedure’s impact on quality of life, an essential consideration in treatment planning.

### 4.1 Limitations

The single-center design may limit the generalizability of the findings to other settings. The relatively small sample size (33 patients completing follow-up) and the high dropout rate (17.5%) may have introduced selection bias, as patients who did not complete post-procedural assessments might differ systematically from those who did. Fertility-focused research, including ovarian reserve assessment and pregnancy outcomes post-UAE, is essential to guide clinical decision-making for younger patients; however, in this study, no fertility markers were collected for analysis. This study did not carry out further subgroup analysis, which might have provided valuable comparisons with previous research. Additionally, investigations should explore systematically the impact of radial access on post-procedural recovery and patient mobility. Lastly, efforts to develop evidence-based pain management protocols and accepted international guidelines will be critical in improving patient experiences and outcomes following UAE.

## 5 Conclusions

UAE has statistically significantly improved symptom severity and health-related quality of life in patients with symptomatic fibroids. PES occurred in 39% of patients, and in the majority of them, it resolved within 2 weeks. Pelvic pain scores improved drastically. 88% of the population was satisfied with UAE results, and 66.7% of patients did not feel the need for further treatments in the follow-up. Future research should prioritize randomized controlled trials to confirm these findings, explore fertility-related outcomes, analyze the effectiveness of radial puncture access in the post-procedural management, and assess peri-procedural pain care, to maximize patient satisfaction and clinical efficacy.
